# Crystallization of Copper Films on Sapphire Substrate for Large-Area Single-Crystal Graphene Growth

**DOI:** 10.3390/nano13101694

**Published:** 2023-05-22

**Authors:** Maxim Komlenok, Pavel Pivovarov, Alexey Popovich, Vladimir Cheverikin, Alexey Romshin, Maxim Rybin, Elena Obraztsova

**Affiliations:** 1 Prokhorov General Physics Institute of the Russian Academy of Sciences, St. Vavilova 38, Moscow 119991, Russia; p_pivovarov@hotmail.com (P.P.); lex@nsc.gpi.ru (A.P.); rybmaxim@gmail.com (M.R.); elobr@kapella.gpi.ru (E.O.); 2 Department of Physical Metallurgy of Non-Ferrous Metals, National University of Science and Technology “MISiS”, Leninskiy Avenue 4, Moscow 119049, Russia; mcm@misis.ru

**Keywords:** copper thin films, copper crystallization, copper annealing, cvd synthesis, graphene monolayer, grain

## Abstract

Chemical vapor deposition synthesis of graphene on polycrystalline copper substrates from methane is a promising technique for industrial production and application. However, the quality of grown graphene can be improved by using single-crystal copper (111). In this paper, we propose to synthesize graphene on epitaxial single-crystal Cu film deposited and recrystallized on a basal-plane sapphire substrate. The effect of film thickness, temperature, and time of annealing on the size of copper grains and their orientation is demonstrated. Under optimized conditions, the copper grains with the (111) orientation and a record size of several millimeters are obtained, and the single-crystal graphene is grown over their entire area. The high quality of synthesized graphene has been confirmed by Raman spectroscopy, scanning electron microscopy, and the sheet resistance measurements by the four point probe method.

## 1. Introduction

To obtain single-layer graphene of a large area and high quality by chemical vapor deposition (CVD), in addition to the synthesis process itself, the surface on which the nucleation of the carbon film begins is important. One of the most efficient ways to grow an ideal graphene monolayer is to use a smooth single-crystal bulk copper [1,2,3]. However, this approach is technologically difficult to implement and scale since it requires the purchase of expensive single-crystal copper. Another already widely used method for the synthesis of graphene is the use of cheap copper foil. The synthesis of high-quality single-layer graphene on a metal foil demands high requirements for the substrate roughness and crystal structure. The foils usually contain some impurities and have a relatively rough surface. Impurities from the surface and the bulk can be removed by using a solid cap during annealing, which acts as a sink for them and leads to an increase in copper purity throughout the catalyst [4]. The coarse surface needs additional processing before the synthesis of graphene to reduce the roughness for the high-quality single-layer graphene growth [5,6]. For this purpose, different methods can be applied such as physical polishing [7], chemical etching [8], annealing [1,9,10], electrochemical polishing [11], and resolidification or liquid metal catalysts [12]. The main advantages of liquid metal over solid substrates are their atomically flat isotropic surface and absence of different defects, such as dislocations, atomic terraces, and impurities [13,14]. Despite recent advances in the real-time monitoring of graphene growth on liquid copper [15], the use of high temperatures and difficulties in the control of the synthesis still limit the wide use of this method for graphene production.

It has been known that the crystalline orientation of Cu substrates plays a crucial role in CVD synthesis of high-quality graphene [16]. In particular, the Cu (111) surface, showing a minimum lattice mismatch of 3.8% with graphene, is expected to provide an ideal catalytic reactivity that reduces defect density, induces larger single-crystalline domain sizes of graphene, and enhances the electrical properties. Therefore, an additional problem that arises from using the foil is the need to control the presence of oxygen on the surface of annealed copper in order to obtain its recrystallization with the desired (111) orientation since the oxidation of the copper surface tends to stabilize (001) orientation [17,18]. A single-crystal Cu (111) substrate can be obtained by using an abnormal grain growth method through the annealing of polycrystalline copper foil in an H_2_ and Ar gas atmosphere [19]. Another approach to obtaining a single crystal graphene on a polycrystalline copper foil is its recrystallization by the mechanical stretching of the foil during annealing [20,21,22].

Against the background of solving the listed problems of copper foil, the synthesis of the high-quality graphene on smooth surface of a copper film, deposited on a polished basal-plane sapphire substrate (α-Al_2_O_3_ (0001)), becomes more urgent [23]. The crystalline structure of the sapphire substrate determines the direction of copper recrystallization during its growth, and its post-annealing. In this case, the large-area single-layer graphene can be grown on epitaxial single-crystal Cu (111). Usually, magnetron radio-frequency sputtering is used for Cu deposition on a sapphire substrate, and the average grain size does not exceed 100 µm [24,25,26,27]. Reddy et al. used thermal evaporation for this purpose and found that epitaxial growth occurs in a narrow substrate temperature range from 240 to 300 °C and that the maximum epitaxial film thickness is limited to ~2 µm [24]. Ma et al. studied the effect of sapphire temperature during deposition on the Cu grain size and the quality of synthesized graphene [28].

The motivation for this study is also the fact that the recrystallized copper film on sapphire, in addition to the obvious prospects in the synthesis of monocrystalline graphene, has a very important and promising application. Such a layered structure consisting of graphene on a thin metal film and transparent substrate can be used for the blister-based laser-induced forward transfer (BB-LIFT) of high-quality graphene patterns to an arbitrary substrate immediately after its synthesis [29,30]. The standard procedure for transferring graphene and forming the necessary pattern from it consists of several stages [31], including the coating graphene with a polymer and its further removal, which introduces a wide range of structural defects into it. Within this context, the BB-LIFT method, having great flexibility in setting the parameters of laser exposure and in the formation of the geometry of the transferred spot, in combination with the already-formed blister system, provides undeniable advantages in comparison with other mechanical and chemical methods of transporting graphene to the final functional surface.

Here, we have separately studied the processes of copper film formation and its recrystallization, as well as their impact on the quality of grown graphene. First, the thermal evaporation of copper is used for the film deposition on sapphire substrate. Then, the thermal annealing in a hydrogen atmosphere is utilized for the film recrystallization. We investigate the effect of film thickness, temperature, pressure, and time of annealing on the size and recrystallization of copper grains and how it affects the quality of grown graphene.

## 2. Materials and Methods

The copper film was deposited on a single-crystal sapphire substrate in a UNIVEX 300 vacuum chamber at low pressure (10^−5^ mbar) when copper pieces (99.999%, ZhongNuo Advanced Material, Beijing, China) were heated and evaporated from a molybdenum boat by passing an electric current through it. The sapphire substrate was washed with potassium dichromate, deionized water and isopropyl alcohol, and annealed at 580 °C in air to clean the surface before the copper deposition. The cleaned substrate was placed in a vacuum chamber and heated to a temperature of 150 °C to remove adsorbed gases and water from the surface. The molybdenum boat with copper pieces was heated up to 2000 °C and the copper evaporated and sputtered on the target substrate at a pressure of 10^−5^ mbar. The final thickness (d) of the copper film varied from 1 to 3.9 µm, depending on the mass of copper pieces in the molybdenum boat. After deposition, the metal film was scratched and its thickness was measured by an interference microscope (Zygo New View 5000, ZYGO Corp., Middlefield, CT, USA).

Before the graphene synthesis, the copper usually was annealed in a hydrogen atmosphere to remove natural oxide from its surface. We used this stage for copper recrystallization. The annealing of copper films on sapphire substrates was carried out at different temperatures (T, from 1000 to 1050 °C) and times (t, 90 and 180 min), in a hydrogen atmosphere (10 mBar). These parameters are the key points for copper film recrystallization and grain formation.

We used a standard tube furnace for CVD graphene growth, and the main feature of the synthesis was the absence of gas flow with optimized gas concentration, pressure, and temperature [32]: pressure 10 mbar, a gas concentration of H_2_:CH_4_ = 10:1, and temperature 1000 °C for 2 min. This simple approach for the realization of the CVD method gives an opportunity to control the easy fabrication of graphene. The quality of graphene was investigated using scanning electron microscopy (SEM, TESCAN Mira 3, TESCAN ORSAY HOLDING, Brno, Czech Republic) and Raman scattering (Horiba LabRAM HR Evolution). In addition, the high quality of the synthesized graphene was confirmed by the sheet resistance measurements performed by the Jandel RM3000 unit using the four point probe method. The distance between two adjacent probes was 1 mm. Raman spectra were recorded with a 532 nm excitation wavelength using 25% of a 20 mW laser focused on the surface by ×100 optical objective after the transfer of graphene from the sapphire/copper film sandwich to a SiO_2_/Si substrate utilizing standard “wet” transfer technology [33]. Raman mapping was carried out on a large sample area of 300 × 400 µm^2^ with a piezoscanner step of 10 µm. In this case, the beam was focused by an ×50 objective into a spot about 2 µm in diameter. For the transfer of graphene from the copper film to a silicon substrate, polymethylmethacrylate (PMMA, 4% in anisole) was applied to the copper film by the spin-coating method at 3000 rpm for 45 s. Then, the copper film was etched by ammonium persulfate ((NH_4_)_2_S_2_O_8_), and PMMA/graphene film was washed with deionized water and transferred onto the silicon substrate. Finally, PMMA was removed with acetone. The surface morphology and grain orientation of the copper films were examined by an optical microscope (Axiotech 25HD, Carl Zeiss, Jena, Germany) and an SEM with electron backscattering diffraction (EBSD) analysis using the NordlysMax2 detector (Oxford Instruments Advanced AZtec Energy, Abingdon, UK). The mean angular deviation (MAD) was 0.1.

## 3. Results and Discussion

**Figure 1:**
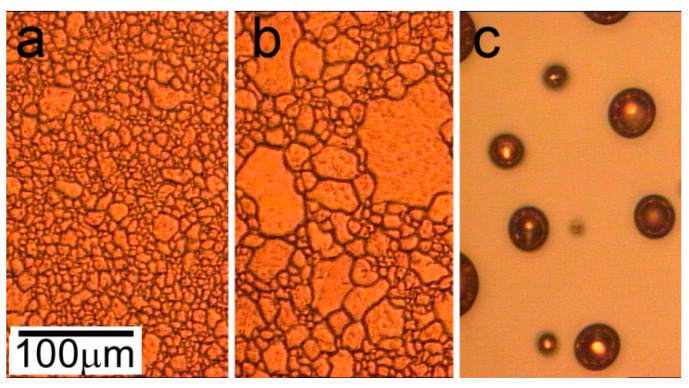
The optical images of copper film with thickness of 3 μm annealed at pressure P = 10 mBar and for t = 90 min at different temperatures: (**a**) T_1_ = 1000 °C; (**b**) T_2_ = 1030 °C; and (**c**) T_3_ = 1050 °C.

The main parameters (t and T) of thermal annealing and cooper film thickness (d) were varied in our experiments to determine the optimal conditions of copper recrystallization, the formation the grains larger than 100 µm, and the synthesis of the graphene layer. First, we fixed two of three parameters (d = 3 µm and t = 90 min) and varied only the temperature in the range of 1000–1050 °C, which is close to the melting point of bulk copper of 1085 °C [34] and is the most critical parameter. The result of film annealing was analyzed with optical microscopy and presented in Figure 1. With an increase in the temperature from 1000 to 1030 °C, the average grain size grows from 10 to 40 µm. A further temperature increase up to 1050 °C leads to the formation of separated drops with an average diameter of 30 µm (Figure 1c) because of the melting-dispersion process occurring in a thin film on an inert sapphire substrate. It involves several competing physical and chemical processes such as local melting; shape optimization resulting from surface area reduction; crystallization; and the melting of the remaining film. The observed melting point for the copper film is slightly lower than the experimental melting temperature for bulk material, which can be explained by the film thickness of microscale size. So, the temperature increase provokes the formation of larger grains until the melting point of a thin metal film is reached.

**Figure 2:**
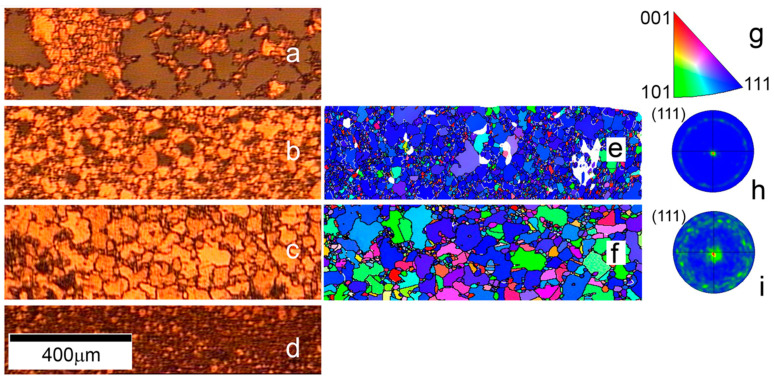
The optical images of copper film with different thicknesses annealed at fixed temperature T = 1030 °C and time t = 90 min: (**a**) d_1_ =1.1 μm; (**b**) d_2_ =1.2 μm; (**c**) d_3_ =2.7 μm; and (**d**) d_4_ =3.9 μm. EBSD map of the copper film obtained from areas corresponding to optical images: (**e**) d_2_ = 1.2 μm; (**f**) d_3_ = 2.7 μm. Inverse pole figure corresponding to EBSD data (**g**); Cu (111) pole figures for the copper film with thickness d_2_ = 1.2 μm (**h**) and d_3_ = 2.7 μm (**i**).

In the next step, having fixed the values of the operating temperature T = 1030 °C, we investigated the influence of the metal film thickness on the size and recrystallization of copper grains at a constant time of annealing t = 90 min. Figure 2a–d present the optical images of copper film with different thicknesses after the annealing procedure. The study of the influence of the film thickness on the process of its recrystallization during annealing revealed the presence of an optimal value. Under the selected annealing conditions, the thinnest film (d = 1.1 μm) becomes torn. An increase in thickness by 100 nm results in more homogeneous domains with an average grain size of 30μm and some gaps between them. The use of the film with d = 2.7 μm induces the formation of grains with an average size of 120 μm. However, a further increase in film thickness to 3.9 μm does not lead to an increase in grain size; on the contrary, a decrease in the size to 10 μm is observed. Apparently, in the last case, the annealing conditions (T, t) could not cause copper recrystallization throughout the entire film thickness. This is also indicated by the maximum grain size of about 40 µm, which is noticeably smaller than 100 µm, at T = 1030 °C in experiments with a varying temperature (Figure 1b) and a fixed film thickness of d = 3 µm. Our experiments have shown that the effect of a slight (~0.1 µm) change in thickness is noticeable only during the transition from fragmented film to the stable recrystallization of copper for thin films with thicknesses of ~1 µm. The changes in the grain size for thicker films occur smoothly with thickness variation under the same annealing conditions. The maximum grain size is observed for the films with a thickness in the range 2.3 to 2.7 µm.

To analyze the crystal texture of copper films after the annealing at different thicknesses, we performed EBSD measurement. This technique showed that crystallites with the (111) orientation are predominantly formed in all annealed copper films (Figure 2e,f). However, there are particulars to be discussed. The annealing of the thin film with d = 1.2 µm at T = 1030 °C and t = 90 min provokes the formation of grains predominantly with the (111) orientation (Figure 2e). The corresponding Cu (111) pole figure presented in Figure 2h shows six Cu (111) poles that are separated by 60° relative to each other in the azimuthal direction. It also shows several weaker and wider Cu (111) poles rotated relative to the stronger poles. These weaker poles have a greater spread in the orientation distribution in the azimuthal direction and correspond to the crystal domains with smaller size and different orientation, scattered over the entire map. The increase in film thickness to d = 2.7 µm leads to an increase in the grain size, but not all of them are oriented along the direction [111] (Figure 2f), and the corresponding (111) pole figure shows a ring pattern (Figure 2i). These data indicate the polycrystalline nature of the copper film. The possible explanation is that in this case the time of annealing is insufficient for the film recrystallization. We can conclude that the film thickness affected the size and orientation of copper grains: the thicker the film, the larger the crystallite size if the temperature and time of annealing are sufficient for copper recrystallization.

**Figure 3:**
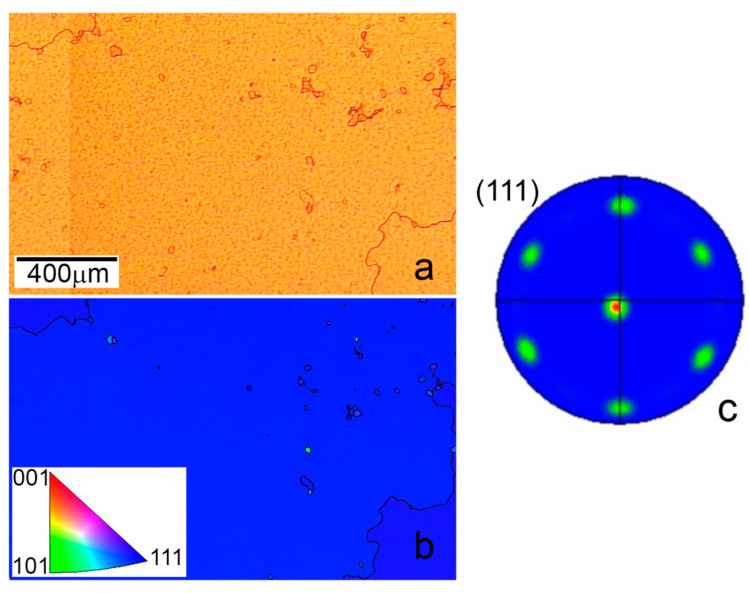
Images of surface of the copper film with thickness d =2.4μm annealed at temperature T = 1030 °C and time t = 180 min: the optical image (**a**), EBSD map with inverse pole figure in inset (**b**), and Cu (111) pole figure (**c**).

Further, for better film recrystallization, we chose the optimal temperature as T = 1030 °C and film thickness d = 2.4 µm and increased the time of thermal annealing to 180 min. The optical image of the copper film annealed under such conditions is shown in Figure 3a. The size of copper grain noticeably increases and exceeds 1 mm in this case. At the same time, diagnostics using EBSD measurements show that the longer annealing time of the film with the thickness close to optimal value, determined in the previous experiment, provides the formation of grains completely (111) oriented (Figure 3b), which is also confirmed by the (111) pole figure showing only six strong Cu (111) poles spaced 60° apart from each other in the azimuth direction (Figure 3c). To the best of our knowledge, such large single-crystal copper grains have not yet been grown on c-plane sapphire. Hu et al. obtained copper grains along the (111) orientation with a two-domain structure and a size not exceeding 20 µm [25] after film (500 nm thickness) annealing for 60 min at 1000 °C. A similar result was demonstrated by Ma et al. [28] after the copper film (750 nm thickness) deposition on the sapphire substrate heated at 623 K.

**Figure 4:**
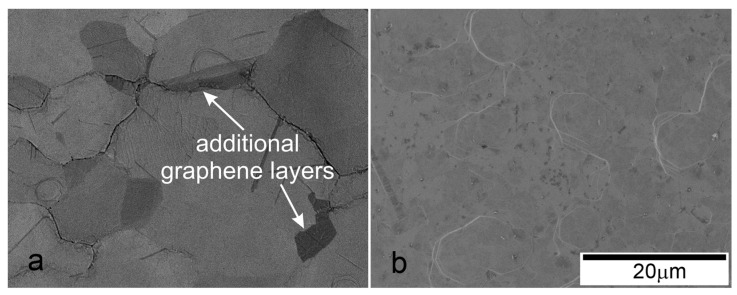
SEM images of graphene synthesized on copper films with thickness: (**a**) d = 1.2 μm; (**b**) d = 2.4 μm.

In the next stage, the CVD synthesis of graphene was carried out on both copper films (preliminarily annealed under the found optimal and non-optimal conditions) in the same tube furnace at an optimized gas concentration. The SEM images of graphene grown on the Cu films with thicknesses d_1_ = 1.2 μm annealed at T = 1030 °C and t = 90 min and d_2_ = 2.4 μm annealed at the same temperature but longer time t = 180 min are shown in Figure 4a,b, respectively. Large dark islands in the image corresponding to additional graphene layers are observed only in Figure 4a along the boundaries between grains. Most of the impurities on the surface of the copper film are also located on the grain boundaries [5]. For the film with large grains, no dark islands with non-single-layer graphene are observed.

**Figure 5:**
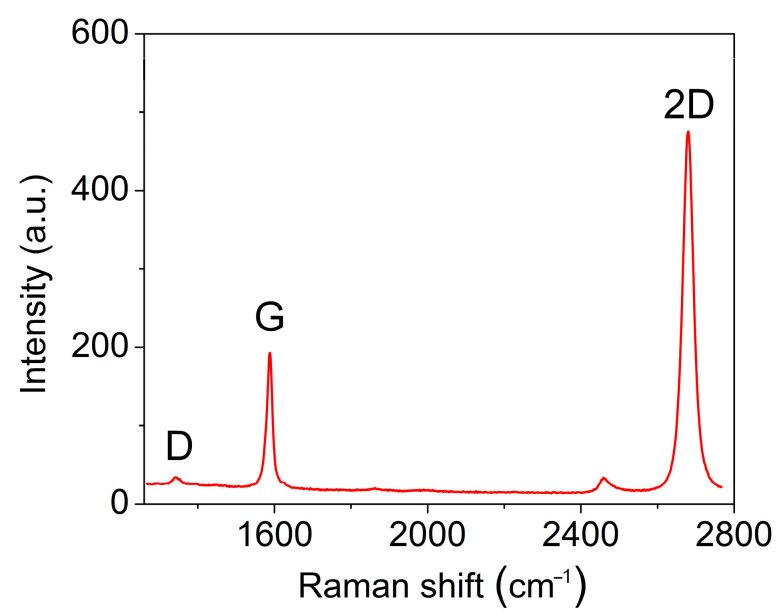
Raman spectra of graphene synthesized on copper film with thickness d = 2.4 μm.

**Figure 6:**
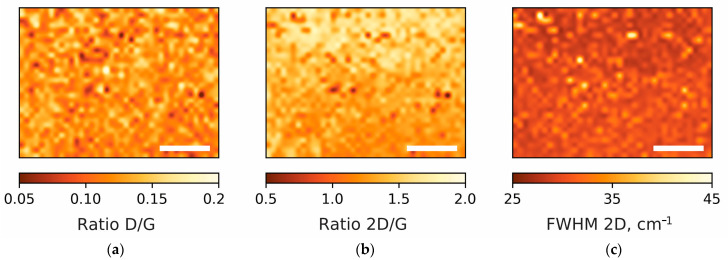
Raman mapping of the three spectra parameters on the area of 300 × 400 µm^2^ with a resolution of 10 µm: (**a**) the D/G peak intensity ratio, (**b**) the 2D/G peak intensity ratio, and (**c**) the 2D peak FWHM. The bar corresponds to 100 µm.

**Figure 7:**
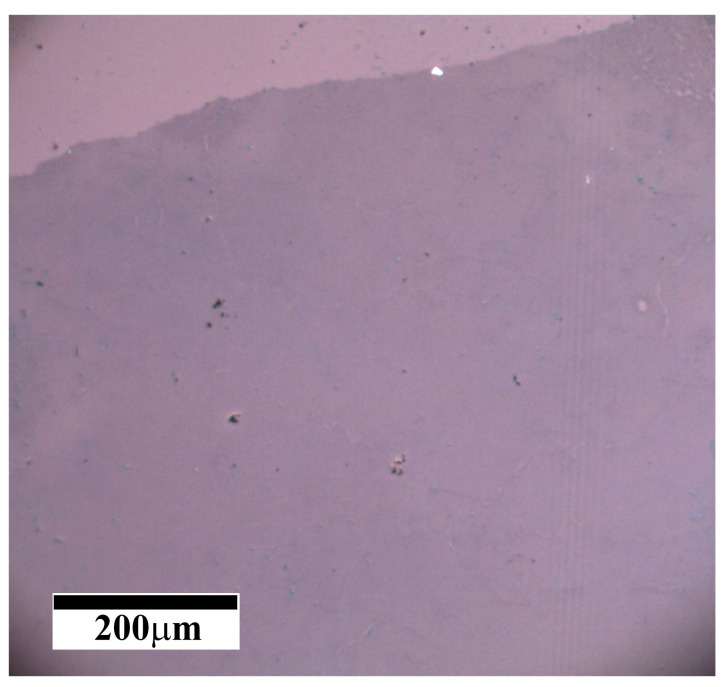
Optical image of graphene synthesized on copper film with thickness of 2.4 μm and transferred onto Si/SiO_2_ substrate.

The quality of the synthesized on Cu (111) film graphene is approved by the Raman spectroscopy recorded in numerous locations after the wet transfer of graphene from the copper film to a SiO_2_/Si substrate. Figure 5 shows a typical spectrum of graphene with three main peaks: the D-peak in the region of 1350 cm^−1^ is responsible for the imperfection of the graphene film or for the distance between defects in the atomic structure; the G-peak in the region of 1580 cm^−1^ is responsible for sp^2^ hybridization in the carbon film and characterizes the high ordering of carbon atoms in the graphene structure; and the 2D peak in the region of 2700 cm^−1^ is the second order of the D-peak, determined by the resonant two-phonon transition, and characterizes the quality of the graphene film in terms of the orderliness and defectiveness of the atomic structure. To determine the orderliness of the atomic structure of a graphene film, one should look at three parameters in the Raman spectra: (1) the intensity ratio of the D/G peaks; (2) the intensity ratio of the 2D/G peaks; (3) and a peak width at half maximum intensity of 2D. Despite the correlation of these three parameters, in any case, when analyzing the quality of the atomic structure of graphene, it is necessary to consider all three parameters and draw conclusions about the quality of the material precisely from the totality of the three values in the characteristics of the Raman spectrum. Thus, when analyzing the synthesized samples, Raman spectra were taken from a large sample area of 300 × 400 µm^2^ with a resolution of 10 µm and a spectrum acquisition area of at least 2 µm. Figure 6 shows the mapping of the three main parameters of the Raman spectra: the D/G peak intensity ratio, the 2D/G peak intensity ratio, and the 2D peak full width at half maximum (FWHM). Figure 6a shows that the ratio of the intensities of the D/G peaks is no more than 0.2, and the average value was calculated as 0.12 ± 0.02. Further, the intensity ratio of the 2D/G peaks varies from 0.5 to 2, with an average value of 1.37 ± 0.19; the width of the 2D peak changes from 25 to 45, and the average value is 30 ± 2 cm^−1^. Despite the fact that the intensity ratio of the 2D/G peaks is considered to be the most important and it is commonly believed that the 2D peak in a graphene monolayer should be 2 times or more higher than the G peak, this is not entirely correct since the peak intensities show the probability of the photons–electron–phonon interaction. The FWHM of the 2D peak is the most revealing parameter to see the difference between single-layer and two-layer graphene; since only two phonons participate in single-layer graphene, there can be only one transition, which is observed in the Raman spectrum as a single Lorentzian peak with width from 20 to 40 cm^−1^. While four phonons are involved in two-layer graphene and there can be four different transitions and a superposition of four individual peaks is observed in the Raman spectrum, the width of the envelope of four peaks cannot be less than 60 cm^−1^ [35,36]. Thus, in Figure 6c (FWHM map) it can be seen that all of the points in the scanned area have a peak width of less than 60 cm^−1,^ and this indicates the complete absence of the second layer in the sample. Moreover, the low ratio of the intensities of the D/G peaks (in Figure 6a, the ratios are no more than 0.2 in the entire region) confirms the high quality of graphene with a distance between defects of more than 25 nm [37,38]. All of these parameters indicate the high quality of the grown graphene over the entire surface of recrystallized Cu film. The optical image of the transfer onto a SiO_2_/Si substrate graphene is presented in Figure 7. The optical contrast of the image allows to see that the graphene film uniformly covers the entire area without the formation of any additional layer, which should be observed as a dark spot. Additional measurements of the sheet resistance of the graphene sample synthesized under optimal conditions have shown values of about 900 Ω/square, which also indicates the high quality of the synthesized graphene at the macrolevel since the distance between two adjacent probes is 1 mm. The measured resistance value is in the range of 426 [39] to 1320 Ω/square [40], measured previously for high-quality single-layer graphene.

## 4. Conclusions

In conclusion, we have optimized the parameters of copper film recrystallization on basal-plane sapphire and increased the grain size by 2 orders of magnitude in comparison with previous works for large-area single-crystal graphene growth. Optimization is achieved by varying the copper film thickness, time, and temperature of its annealing. This make it possible to recrystallize a copper grain a few millimeters in size oriented along the [111] direction, which is optimal for graphene synthesis, during the usual stage of substrate annealing in a hydrogen atmosphere to remove natural oxide from its surface before graphene growth. In this case, high-quality single-crystal graphene is synthesized over the entire surface of recrystallized copper film. The quality of graphene has been approved by Raman spectroscopy, scanning electron microscopy, and the sheet resistance measurements using the four point probe method. The obtained result demonstrates the high potential of graphene CVD synthesis on thin copper films for industrial production and application. Of particular interest, in our opinion, is the possibility of using such a sandwich (sapphire/copper film/graphene) for the BB-LIFT of graphene patterns on an arbitrary substrate.

## Data Availability

Data can be made available upon request from the authors.
